# Peroral pancreatoscopy-guided lithotripsy via an endoscopic ultrasonography-guided pancreatogastrostomy

**DOI:** 10.1055/a-2445-8419

**Published:** 2024-11-13

**Authors:** Masatoshi Murakami, Nao Fujimori, Akihiko Suenaga, Yumeka Kawaguchi, Akihisa Ohno, Kazuhide Matsumoto, Keijiro Ueda

**Affiliations:** 1 Department of Medicine and Bioregulatory Science, Graduate School of Medical Sciences, Kyushu University, Fukuoka, Japan

A 72-year-old man with autoimmune pancreatitis type 2 presented with acute pancreatitis due to an impacted pancreatic stone ( Fig. 1 ). Immediate transpapillary drainage was unsuccessful, leading us to attempt drainage via endoscopic ultrasonography-guided pancreaticogastrostomy (EUS-PGS) ( Video 1 ).

**Fig. 1:**
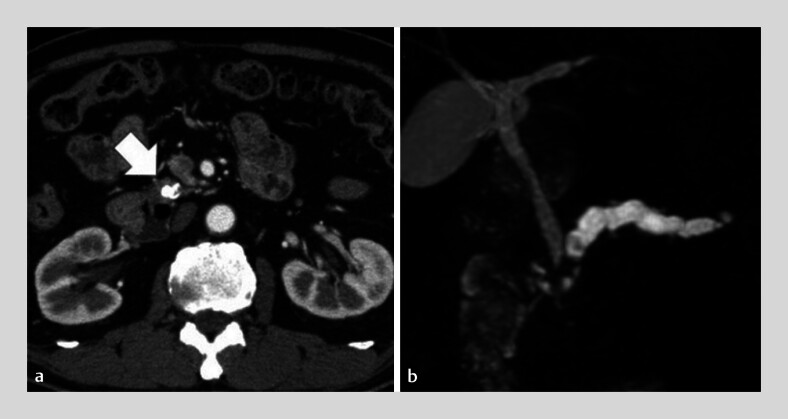
Imaging studies. **a** Computed tomography scan showing multiple pancreatic stones, with a maximum size of 10 mm (arrow), in the pancreatic head. **b** Magnetic resonance imaging revealing a dilated main pancreatic duct, with a maximum diameter of 8 mm.

Endoscopic ultrasonography-guided pancreaticogastrostomy for stone removal and stent placement.Video 1

A linear echoendoscope (EG-740UT; Fujifilm, Tokyo, Japan) was used to puncture the pancreatic duct with a 19-G fine-needle aspiration needle (EZ Shot 3 Plus; Olympus, Tokyo, Japan). The guidewire was successfully advanced into the duodenum using a 3-Fr microcatheter (Daimon ERCP catheter; Hanaco Medical, Saitama, Japan), which improved guidewire control. After the punctured tract over the stones had been dilated with a drill dilator (Tornus ES; Asahi Intecc, Aichi, Japan), a plastic stent (TYPE-IT; Gadelius Medical, Tokyo, Japan) was placed ( Fig. 2 **a** ).

**Fig. 2:**
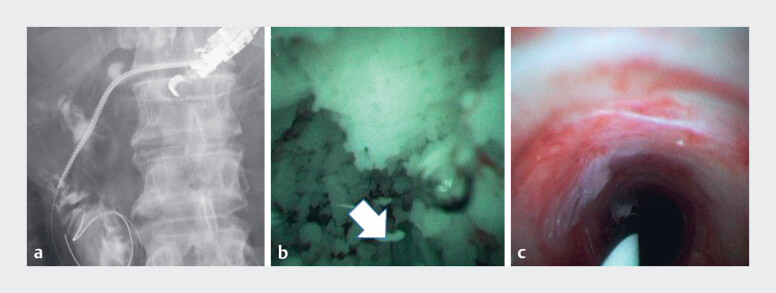
Stone fragmentation and removal. **a** Easy dilation of the puncture tract over the stone using a drill dilator (Tornus catheter; Asahi Intecc, Aichi, Japan), despite challenges with endoscopic retrograde cholangiopancreatography and balloon catheters. **b** Electrohydraulic lithotripsy fragmentation and stone removal by pushing the peroral pancreatoscope into the duodenum (arrow; electrohydraulic lithotripsy probe). Procedures were performed using a 9-Fr peroral pancreatoscope (eyeMAX; Micro-Tech, Nanjing, China). **c** Endoscopic confirmation of minimal residual stones.

One month later, a 9-Fr peroral pancreatoscope (POPS) (eyeMAX; Micro-Tech, Nanjing, China) was inserted via the EUS-PGS, and the stones were fragmented using electrohydraulic lithotripsy (EHL; Nortech AUTOLITH lithotripter with a 1.9-Fr probe; Northgate Technologies, Illinois, USA). Following endoscopic pancreatic sphincterotomy using a rendezvous technique, the fragmented stones were completely crushed transgastrically and removed by pushing the POPS through the papilla ( Fig. 2 **b, c** ). Due to obstruction of the papilla by the fragmented stones, stent placement via the EUS-PGS was not possible. The stones were therefore removed endoscopically using grasping forceps, and the stent was pulled out from the papilla under fluoroscopic guidance to secure the EUS-PGS route. The patient remained asymptomatic 2 months after the intervention.

Recently, numerous procedures have been conducted via EUS-PGS 1 , and the efficacy of transgastric POPS with EHL has been documented 2 3 4 . In the current case, the stones were successfully removed by propelling the POPS through the papilla into the duodenum, in conjunction with conventional endoscopic retrograde cholangiopancreatography techniques. Stone removal using POPS via EUS-PGS could potentially be a valuable treatment option for challenging stones.

Endoscopy_UCTN_Code_TTT_1AR_2AI

## Notes

Endoscopy E-Videos https://eref.thieme.de/e-videos
*E-Videos* is an open access online section of the journal *Endoscopy* , reporting on interesting cases and new techniques in gastroenterological endoscopy. All papers include a high-quality video and are published with a Creative Commons CC-BY license. Endoscopy E-Videos qualify for HINARI discounts and waivers and eligibility is automatically checked during the submission process. We grant 100% waivers to articles whose corresponding authors are based in Group A countries and 50% waivers to those who are based in Group B countries as classified by Research4Life (see: https://www.research4life.org/access/eligibility/ ). This section has its own submission website at https://mc.manuscriptcentral.com/e-videos .
